# Specialties in Dentistry

**Published:** 1864-11

**Authors:** C. M. Wright


### SPECIALTIES IN DENTISTRY.

Read before the Cincinnati Dental Society.

BY DR. C. M. WRIGHT.

Dentistry, which is itself a specialty of medicine, is, in my opinion capable of being divided into *at least* two departments—the operative and mechanical. This is no new opinion, nor is it a new subject, It has been talked of and written about for some time by members of the profession; but no decided movement has been made, I believe, in this neighborhood by intelligent dentists to create the specialties of operative and mechanical dentistry. To a certain extent in all offices in cities one of these branches has been cultivated at the expense of the other, or rather, nearly every dentist is considered as either an operator or a mechanic. One has a mechanical business, another an operative, and again another has a large proportion of the diseases of the mouth to attend to. The same fact is noticed in mechanical men's practice. A certain kind of work as continuous gum or vulcanite or gold plate is demanded, in the office where this certain style of work is made *rather* a specialty. This may be taken as part proof that the tendency of an improving skill in, and an increasing knowledge of, dentistry, is toward a division of the practice, and that the time has arrived when our branch of the great healing art may be profitably subdivided into at least the operative and mechanical. The good old-fashioned country physician who carried in his saddle-bags and brains the means and the skill for attending to all the "ills that flesh is heir to," would no doubt have laughed at the idea of a class of practitioners who would take the eye or the ear, or the lungs for their province, and devote their whole lives to the study and cure of so small a part of the human body. And there are now no doubt "tooth doctors," who agree "to perform *all* operations on the teeth in the latest and most approved styles," and to warrant them at that; that would ridicule the man who could find enough in one department of dentistry, to employ all his energies for its advancement. In the country, where many believe that filling "rots the teeth," and where others will "gum it," rather than afford the cost of the new-fangled notion, a set of artificial teeth, one pair of hands is expected to do all it can for the good of the patients it is fortunate enough to secure. In cities it is different, and it seems to me, that it would be a long step towards the perfection of our profession if the two departments were made specialties. Then there are men who have decided talents and strong tastes for one or the other of these branches. One has a genius for filling teeth, for correcting irregularities, etc., etc., the labor of grinding in artificial teeth, of carving in plaster, of working the metals, and of blowing his life away at the blow-pipe, is to him, to say the least, very dull work. He has no taste for it. He can not enlist his soul in his labor. He dreads going into his laboratory; while his neighbor on the other hand trembles when he is called upon to take his hands out of the moulding sand, to perform some delicate operation on the living organs of mastication. The mechanical operator's talents and tastes find ample scope within his boundaries. He studies the appearances and uses of the "pearls" of the mouth, and the powers of expression of the "human face divine," and it is his highest ambition to imitate nature. He can become quite enthusiastic at his lathe, and the feeling and the name of "common mechanic at his trade" belongs not to him. He is an artist, with nature for his model, and chemistry, anatomy, metallurgy, etc., for his instruments. If the operator could confine himself to his chair; if he could have this dread of the laboratory taken from his mind; if he could feel that from this time forth he was a physician and surgeon of the mouth and teeth, I believe his every operation would be better. He would advance his specialty and move onward, right onward in the direction of perfection. His fingers would be better trained for the exceedingly nice manipulation of his art. He would become more a master of his profession, and reputation, and money, and satisfaction would be his. The mechanical dentist too would have better opportunities, and be in a better condition to advance his specialty. By drawing a line between the two department, both would be benefited, and improved; and dentistry as a profession would be advanced. A young man after completing his primary education, could then choose his departments, and if he should devote all his energies to operative dentistry, or to mechanical dentistry is it not very probable that in his life practice, he would be a much more successful operator in his chosen sphere, and would not his specialty—his hobby—be brought to a higher state of cultivation, to a better pace, than though he were expected to perfect himself in all the various and different operations of the whole science and art of preserving, repairing, and replacing the teeth? We can not but think he would, and that if every man should choose his specialty, make one branch his study, devote himself to one department of dentistry, every man would be a better dentist. In this case he himself, the profession and the patients too, would enjoy the advantages of this concentrated labor.

[The discussions upon the subject will be given in the next No,—Ed.]

